# Prevalence and Associated Factors of Glaucoma Among Adults in a Tertiary Care Center in Dubai, United Arab Emirates: A Retrospective Review

**DOI:** 10.7759/cureus.98105

**Published:** 2025-11-29

**Authors:** Maryam Jafari, Sarah Alkabbani, Gunjan Awatramani, Ghazal Talal Saeed, Jeyaseelan Lakshmanan, Sandeep Thakur, Pramod Warhekar

**Affiliations:** 1 College of Medicine, Mohammed Bin Rashid University of Medicine and Health Sciences, Dubai, ARE; 2 Biostatistics and Epidemiology, Mohammed Bin Rashid University of Medicine and Health Sciences, Dubai, ARE; 3 Ophthalmology, Mediclinic City Hospital, Dubai, ARE

**Keywords:** epidemiology, glaucoma, prevalence, risk factors, united arab emirates

## Abstract

Glaucoma is a leading cause of blindness worldwide, with its impact expected to increase substantially in the coming years. It results from structural and functional damage to the optic nerve and can be classified into primary open-angle glaucoma (POAG), primary angle-closure glaucoma (PACG), and secondary glaucoma. This retrospective cross-sectional study is among the first to examine the prevalence and extent of glaucoma in a tertiary care center in the United Arab Emirates (UAE). Adults over the age of 18 who presented to the ophthalmology outpatient clinic between January 2017 and December 2021 with a confirmed diagnosis of glaucoma were analyzed. Data collected included demographic variables such as age, gender, and nationality, as well as comorbidities including diabetes, hypertension, and family history of glaucoma. Data collection resulted in 553 glaucomatous eyes, where the mean age was 53.3 ± 14.0 years (range 20-98 years), and the majority of participants were male (60%, n = 332) compared to 40% of females (n = 221). Looking at the proportion of glaucoma by year, it was evident that 38.1% of eyes were severely affected in 2017, and this proportion increased to 53.9% by 2021. UAE nationals had the highest rate of severe glaucoma (60.7%), in contrast to patients from India (38.6%), the United Kingdom (26.2%), and other nationalities (46.4%) (p < 0.001). There was a significant association between age and severe glaucoma, with prevalence lowest in the youngest group (≤28 years, 25.0%) and highest in the oldest group (≥69 years, 68.6%; p < 0.001). Male gender was also significantly associated with severe disease, whereas diabetes, hypertension, and family history showed no significant associations. To our knowledge, this study is the first of its kind in the UAE that looks into the risk factors and epidemiology of glaucoma. The data from this study will aid in filling the gap with regard to prevalence data in the UAE, which can further guide screening and prevention measures.

## Introduction

Glaucoma is a progressive eye disease characterized by structural and functional damage to the optic nerve and related visual impairment, which can result from various pathological mechanisms [1].

Glaucoma is a significant public health issue, being a leading cause of blindness worldwide, and its prevalence continues to increase. Individuals may be asymptomatic initially, only seeking diagnosis and treatment after considerable irreversible vision loss, making it an even more pressing public health concern [2]. In 2013, an estimated 64.3 million people globally were affected by glaucoma, a number that rose to 76 million by 2020 and is projected to reach 111.8 million by 2040 [3].

Glaucoma can be broadly categorized into subtypes based on the underlying anatomy and pathophysiology. Primary glaucoma consists of the primary open-angle glaucoma (POAG) variant or the primary angle-closure glaucoma (PACG). Secondary glaucoma, on the other hand, is due to an identifiable cause such as trauma, surgery, or medications, to name a few [4].

Major risk factors for this disease include a raised intraocular pressure (IOP), which has traditionally been considered a defining feature, but this idea has largely been abandoned in recent years [5]. Other risk factors include age, race, co-morbidities, and refractive error, as well as family history [6].

The prevalence of glaucoma, its subtypes, and associated risk factors have been extensively studied in several population-based investigations around the world. Findings from African and European studies highlight significant racial differences in glaucoma types, with reports indicating that African American individuals are more susceptible to open-angle glaucoma compared to White individuals [7]. However, the Middle East still lacks comprehensive research to characterize glaucoma in the region [8]. Although studies have been conducted in some Middle East countries such as Iran, Qatar, Oman, and Saudi Arabia [9], no extensive research has yet been reported from the UAE. Investigating risk factors in the Middle East is particularly important considering the region’s multicultural population and the high prevalence of comorbidities such as diabetes [10]. In the United Arab Emirates (UAE), the prevalence of diabetes was 18.7% in 2010 and is projected to climb to 21.4% by 2030, surpassing the global average [11]. With the well-established link between diabetes mellitus and glaucoma, as diabetic patients are significantly more likely to develop glaucoma [12], it becomes more crucial to study the prevalence of glaucoma more extensively in the region.

This retrospective study, to our knowledge, is one of the first attempts to assess the extent and burden of glaucoma in a hospital setting in Dubai. We aimed to describe the distribution and subtypes of glaucoma among adults attending a tertiary care center and to compare demographic and clinical characteristics across severity categories. Given the underexplored literature around glaucoma prevalence in Dubai and the UAE, we aim to explore the epidemiology of glaucoma to guide future public health measures.

This article was previously presented as a meeting abstract at the 6th Annual Mediclinic Middle East Research Conference held on March 16, 2023.

## Materials and methods

This study is a retrospective cross-sectional study with a sample size of adults who presented to the Ophthalmology Department at Mediclinic City Hospital, a tertiary care hospital in Dubai, UAE, between January 2017 and December 2021, with a diagnosis of established “glaucoma” as per their electronic medical record. Patients who visited the glaucoma clinic were of various nationalities, aged above 18 years old, and suffering from comorbid conditions such as diabetes and hypertension.

All patients who were diagnosed with glaucoma over the course of five years were included in the study. The study adhered to the diagnostic and classification criteria established by the International Society for Geographical and Epidemiological Ophthalmology (ISGEO), consistent with methods used in other hospital-based studies.

The sampling method utilized was non-probability time frame sampling, wherein all the patients with a diagnosis of glaucoma who visited the hospital outpatient department were included in the study. All adults who visited the hospital and were diagnosed with glaucoma during the five-year period were included in the study. Patients under the age of 18 years, as well as those who were diagnosed with pre-glaucoma, were excluded from the study. Patients who were diagnosed with ocular hypertension and no changes to the optic nerve were excluded from the study. Additionally, patients with any ocular conditions that could affect the validity of the fundus examination were excluded from the study. These conditions include retinitis pigmentosa, macular degeneration, and diabetic retinopathy, as well as hypertensive retinopathy. Those participants with incomplete records were further excluded.

Data was collected using the electronic medical records system at the hospital to extract specific variables regarding the patient’s established diagnosis of glaucoma. The IT department was able to provide the list of all the patients who were diagnosed with glaucoma in the ophthalmology outpatient department between the years 2017 and 2021. Specific data variables that were extracted from the patients’ files included demographics such as age, gender, and nationality. Information on risk factors, including diabetes, hypertension, and first-degree family history of glaucoma, was extracted from each patient’s electronic medical record based on documented diagnoses and clinical history. With regard to the clinical data variables, the type of glaucoma, the visual acuity, the presenting IOP, the cup-to-disc ratio, the glaucoma hemifield testing, and the scanning laser ophthalmoscopy findings were included.

Visual acuity was initially recorded in Snellen notation and later converted to logMAR notation. IOP was measured using a Goldmann applanation tonometer attached to a slit lamp biomicroscope, following standard procedure. Glaucoma diagnosis involved gonioscopy with a Goldmann 3-mirror lens and a fundus examination using a Welch-Allyn ophthalmoscope (Welch Allyn, Inc., USA). The glaucoma hemifield test (GHFT) was carried out using the automated Humphrey visual field analyzer (Carl Zeiss Meditec, Germany). Glaucomatous optic disc atrophy was verified through stereoscopic examination of the optic disc using a +90D lens on the slit lamp. In addition, data on central corneal thickness (CCT) and retinal nerve fiber layer (RNFL) loss were collected.

Glaucoma was classified into categories based on type, as primary and secondary. Primary was further subclassified into POAG or PACG according to the anterior chamber angle morphology as examined by gonioscopy. Secondary glaucoma was classified when an underlying cause, such as trauma, medication-induced, or inflammatory processes, etc., was attributed to the glaucoma [4].

The severity of glaucoma was then classified into mild, moderate, and severe based on the International Classification of Diseases-10 (ICD-10) glaucoma severity reference guide [13].

The association between sociodemographic variables, baseline morbidities, and severity of glaucoma was studied using the chi-square test. The difference between the means of IOP according to the severity of glaucoma was tested using analyses of variance (ANOVA). All data analysis was performed using the IBM SPSS Statistics for Windows, Version 29 (released 2022; IBM Corp., Armonk, New York, United States).

The Institutional Review Board of the Dubai Scientific Research Ethics Committee (DSREC), Dubai Health Authority, had provided approval for this project (DSREC-01/2022_10), which adheres to the principles of the Declaration of Helsinki. Permission to access the electronic medical records was also obtained from the management of the Mediclinic City Hospital.

## Results

Data collection initially resulted in 1,106 glaucomatous eyes from patients attending our tertiary care ophthalmology clinic. In total, 553 patients had complete demographic and clinical data available; however, complete data records for all variables required for severity grading were found in only 494 eyes. The mean age was 53.3 ± 14.0 years (range: 20-98 years). The majority of participants were male (60%, n = 332) compared to 40% of females (n = 221).

The most common type of glaucoma was POAG, with a rate of 58.2% (95% CI: 55%-61%); this was followed by secondary angle glaucoma at 29.7% (Table 1).

**Table 1:** Baseline demographic, systemic, and ocular characteristics of the study population (N = 553 patients) Data are presented as n (%) for categorical variables and mean ± SD for continuous variables. IOP: intraocular pressure

Variable	Category	N (%)	%
Gender	Male	332	60.0%
	Female	221	40.0%
Nationality	UAE	92	16.6%
	India	160	28.9%
	UK	45	8.1%
	Others	256	46.3%
Comorbidities	Diabetes	110	19.9%
	Hypertension	96	17.4%
Family History of Glaucoma	Present	30	5.4%
	Absent	523	94.6%
Mean Age		53.3 ± 14.0 years	
Type of Glaucoma	Primary Open Angle	322	58.2%
	Secondary	164	29.7%
	Primary Angle Closure	60	10.8%
	Normal Tension	7	1.3%
Visual Acuity (LogMAR)	Right Eye	0.8 ± 0.7	
	Left Eye	0.8 ± 0.8	
IOP (mmHg)	Right Eye	18.2 ± 6.7	
	Left Eye	18.6 ± 6.6	
Cup-to-Disc Ratio	Right Eye	0.54 ± 0.16	
	Left Eye	0.54 ± 0.16	
Pachymetry (µm)	Right Eye	540.9 ± 44.7	

The severity of glaucoma was then classified into mild, moderate, and severe based on the ICD-10 glaucoma severity reference guide. Around 206 eyes (41.7%) had mild severity, 71 eyes (14.4%) had moderate severity, and 217 eyes (43.9%) had severe glaucoma (Table 2). It is important to note that data for visual fields were missing for 612 eyes (55.3%), which were excluded from severity classification.

**Table 2:** Distribution of glaucoma severity across demographic, systemic, and clinical parameters (N = 494 eyes) The chi-square test was used for categorical variables and one-way ANOVA (F-test) for continuous variables. Data are presented as n (%) or mean ± SD. VA: visual acuity; IOP: intraocular pressure

Variable	Mild (n = 206)	Moderate (n = 71)	Severe (n = 217)	Test Statistic	p-value
Age Group (years)				χ²= 33.69	<0.001
≤28	8 (66.7%)	1 (8.3%)	3 (25.0%)		
29–38	22 (42.3%)	4 (7.7%)	26 (50.0%)		
39–48	57 (50.4%)	24 (21.2%)	32 (28.3%)		
49–58	64 (43.8%)	23 (15.8%)	59 (40.4%)		
59–68	44 (36.7%)	14 (11.7%)	62 (51.7%)		
≥69	11 (21.6%)	5 (9.8%)	35 (68.6%)		
Gender				χ²= 6.69	0.035
Male	130 (41.5%)	36 (11.5%)	147 (47.0%)		
Female	76 (42.0%)	35 (19.3%)	70 (38.7%)		
Nationality				χ²= 23.22	<0.001
UAE	11 (18.0%)	13 (21.3%)	37 (60.7%)		
India	73 (46.2%)	24 (15.2%)	61 (38.6%)		
UK	24 (57.1%)	7 (16.7%)	11 (26.2%)		
Others	98 (42.1%)	27 (11.6%)	108 (46.4%)		
Diabetes				χ²= 4.20	0.122
Yes	28 (32.2%)	16 (18.4%)	43 (49.4%)		
No	178 (43.7%)	55 (13.5%)	174 (42.8%)		
Hypertension				χ²= 1.96	0.375
Yes	25 (34.2%)	12 (16.4%)	36 (49.3%)		
No	181 (43.0%)	59 (14.0%)	181 (43.0%)		
Family History				χ²= 3.41	0.181
Present	17 (56.7%)	2 (6.7%)	11 (36.7%)		
Absent	189 (40.7%)	69 (14.9%)	206 (44.4%)		
IOP (mmHg)	18.2 ± 5.4	18.7 ± 4.7	18.5 ± 6.8	F= 0.327	0.722
VA (LogMAR)	0.9 ± 0.2	0.9 ± 0.2	0.9 ± 0.2	F= 0.783	0.458
Pachymetry (μm)	533.9 ± 36.5	541.7 ± 38.0	530.9 ± 64.3	F= 0.721	0.487
Cup:Disc Ratio	0.5 ± 0.1	0.6 ± 0.1	0.6 ± 0.2	F= 5.968	0.003

Looking at the prevalence of glaucoma severity by year, we observed a statistically significant variation (p = 0.004). In 2017, 38.1% of eyes were severely affected, and this proportion increased to 53.9% by 2021. As for gender, there was a significant statistical difference between males and females. Severe glaucoma was found in 47.0% of eyes from male patients compared to 38.7% in female patients (p = 0.035).

UAE nationals had the highest rate of severe glaucoma (60.7%), in contrast to patients from India (38.6%), the United Kingdom (26.2%), and other nationalities (46.4%) (p < 0.001). Similarly, age was significantly associated with severe glaucoma. The youngest group (≤28 years) had a 25.0% rate of severe glaucoma, compared to 68.6% among the elderly aged ≥69 (p < 0.001).

No statistically significant difference was found between glaucoma severity and diabetes mellitus, hypertension, or family history of glaucoma.

For clinical parameters, the mean IOP was similar across different severities of glaucoma with no statistical difference (mild: 18.2 ± 5.4 mmHg, moderate: 18.7 ± 4.7 mmHg, severe: 18.5 ± 6.8 mmHg; p = 0.722). Visual acuity and pachymetry values also did not differ significantly among the groups (VA: 0.9 ± 0.2; pachymetry: 533.9-541.7 µm; p = 0.458 and 0.487, respectively). However, the cup-to-disc ratio (CDR) was significantly higher with disease severity (normal/mild: 0.5 ± 0.1, moderate: 0.6 ± 0.1, severe: 0.6 ± 0.2; p = 0.003).

## Discussion

Glaucoma, a chronic and progressive optic neuropathy, remains one of the leading causes of irreversible blindness globally. This cross-sectional study aimed to determine the prevalence of glaucoma among adults in a tertiary center in Dubai, UAE, providing a critical insight into the burden of this potentially blinding disease in a rapidly developing population.

To our knowledge, this study is the first to examine the risk factors and epidemiology of this progressive disease, as the prevalence of glaucoma in the UAE is unknown.

The prevalence of POAG was 58.2%, followed by PACG. The prevalence rate in Dubai is comparable to that reported in neighboring Gulf countries, such as Saudi Arabia and Oman. Specifically, in Saudi Arabia, a retrospective analysis was performed by Helayel et al. and published in 2021, and the three most common types of glaucoma to be found were POAG, which was 27.7%; followed by secondary glaucoma at 26.7%; and finally, primary ACG at 18.2% [14]. The mean age of patients in their study was approximately 59 years old. This is comparable to our study, where the mean age was 53.3 years.

However, it is worth noting that differences exist in the reported prevalence of glaucoma between hospitals in the country. For example, in another hospital in Saudi Arabia, the most common type of glaucoma was primary ACG (46.6%), followed by PAC, secondary glaucoma, and POAG, respectively [15]. This variation could be partly explained by the smaller sample size. In a study by Khandekar et al., involving 940 patients from Saudi Arabia, males were found to have a higher prevalence of glaucoma than females [16]. Similarly, in the UAE, within the tertiary center where our study was conducted, glaucoma prevalence was also higher in males, at 60%. In the Oman Eye Study (2005), glaucoma prevalence among individuals over the age of 30 was reported at 4.75% [17]. More recently, a study by Abu-Shaheen et al., published in May 2025 in Saudi Arabia, found that females had a higher prevalence of glaucoma (54.2%) [18].

Age and gender emerged as significant risk factors in our study, with higher prevalence rates observed among older adults and males. Demographic risk factors such as age and gender can show differences in the prevalence of glaucoma as well. For instance, in central Iran, the ratio of glaucoma between men and women was found to be 37:50 in POAG [9].

These statistics can be compared to other Middle Eastern countries, such as Qatar, where a cross-sectional study was performed on individuals aged 40 years and older [19]. Out of the 3,149 participants enrolled, the overall prevalence of glaucoma was stated to be 1.73%. POAG was found in 65.7% of glaucoma patients, with lower percentages of PACG and other types of glaucoma, such as post-traumatic and pseudoexfoliation. However, this study was performed in 2009, and the updated statistics could show a difference in the prevalence of glaucoma types. The prevalence in males was also found to be higher than in females. 

In terms of the severity of glaucoma, 217 patients had the highest prevalence of severe glaucoma (43.9%). In Saudi Arabia, the most recent study also showed similar results, with 42.5% of their patients suffering from severe glaucoma [18]. The age of the patient was also found to be correlated with the severity of glaucoma, where patients above the age of 69 were found to have the highest prevalence of severe glaucoma at 68.6% in our study. Mild glaucoma was found to be the most prevalent in the younger population ≤28 at 66.7%. Similar trends were found in another study conducted in the UK, where age was found to be a significant factor, with the presence of severe glaucoma seen in elderly patients [20].

Co-morbidities such as diabetes and hypertension were also found to be prevalent in our study population, corresponding to 19.9% and 17.4%, respectively. Moreover, patients with severe glaucoma were found to have the highest prevalence of hypertension (49.3%) and diabetes (49.4%). The Blue Mountains Eye Study, which examined over 3000 patients, suggested that the presence of hypertension, particularly if poorly controlled, can modestly increase the risk of OAG [21]. In another meta-analysis of population-based studies by Bae et al., which included over 60,000 patients, similar patterns were suggested with an increased risk of OAG with systemic hypertension [22].

While this study provides valuable insights, it is essential to acknowledge certain limitations. Approximately half of the initially identified eyes had incomplete records and were excluded, which may introduce missing-data bias. The cross-sectional design precludes the establishment of causal relationships. Furthermore, the study was conducted retrospectively at a single medical institution with the use of convenience sampling, not offering a representation of the larger population in the UAE. As a tertiary referral center, the study population may over-represent more advanced or complex glaucoma cases, limiting generalizability. To be able to appreciate the findings in this study, a larger sample size from varying medical institutions across other Emirates would be required. Given that the hospital was part of the private healthcare sector, it doesn’t take into account the differences in population that might present with glaucoma in the public sector, which could include factors such as socioeconomic status and access to healthcare. There was also a lack of other data parameters, such as the visual field tests, that were pertinent to assessing the effect of glaucoma on vision. The analysis of this additional data could allow us to draw conclusions on the impact of the severity of glaucoma on visual outcomes that may be subtle to the patient or only evident with the help of additional imaging techniques. However, this study would be one of the first studies to delve into the epidemiological characteristics of glaucoma in the UAE, which can be compared to other countries in the Middle East region.

The data from this study will aid in filling the gap with regard to prevalence data in the UAE, which can further guide screening and prevention measures.

## Conclusions

This study offers one of the first insights into the patterns, severity, and risk factors of glaucoma in a tertiary care center in Dubai, UAE. POAG was the most common subtype, and a high proportion of patients presented with severe disease, particularly older individuals and UAE nationals. Age and gender were significant factors, while associations with diabetes and hypertension were less clear, emphasizing the need for larger studies to explore these links further in the local context.

Although limited by its single-center, retrospective design, this study addresses an important gap in the regional literature. The findings highlight the value of early detection and the importance of screening initiatives to reduce the burden of irreversible vision loss. As the prevalence of glaucoma continues to rise globally, generating local data is essential for shaping prevention strategies and guiding healthcare planning in the UAE.
